# Epigenetic Effects Induced by Methamphetamine and Methamphetamine-Dependent Oxidative Stress

**DOI:** 10.1155/2018/4982453

**Published:** 2018-07-22

**Authors:** Fiona Limanaqi, Stefano Gambardella, Francesca Biagioni, Carla L. Busceti, Francesco Fornai

**Affiliations:** ^1^Human Anatomy, Department of Translational Research and New Technologies in Medicine and Surgery, University of Pisa, Via Roma 55, Pisa, Italy; ^2^IRCCS Neuromed, Via Atinense, Pozzilli, Italy

## Abstract

Methamphetamine is a widely abused drug, which possesses neurotoxic activity and powerful addictive effects. Understanding methamphetamine toxicity is key beyond the field of drug abuse since it allows getting an insight into the molecular mechanisms which operate in a variety of neuropsychiatric disorders. In fact, key alterations produced by methamphetamine involve dopamine neurotransmission in a way, which is reminiscent of spontaneous neurodegeneration and psychiatric schizophrenia. Thus, understanding the molecular mechanisms operated by methamphetamine represents a wide window to understand both the addicted brain and a variety of neuropsychiatric disorders. This overlapping, which is already present when looking at the molecular and cellular events promoted immediately after methamphetamine intake, becomes impressive when plastic changes induced in the brain of methamphetamine-addicted patients are considered. Thus, the present manuscript is an attempt to encompass all the molecular events starting at the presynaptic dopamine terminals to reach the nucleus of postsynaptic neurons to explain how specific neurotransmitters and signaling cascades produce persistent genetic modifications, which shift neuronal phenotype and induce behavioral alterations. A special emphasis is posed on disclosing those early and delayed molecular events, which translate an altered neurotransmitter function into epigenetic events, which are derived from the translation of postsynaptic noncanonical signaling into altered gene regulation. All epigenetic effects are considered in light of their persistent changes induced in the postsynaptic neurons including sensitization and desensitization, priming, and shift of neuronal phenotype.

## 1. Introduction

### 1.1. Molecular Mechanisms of Methamphetamine

**Figure 1:**
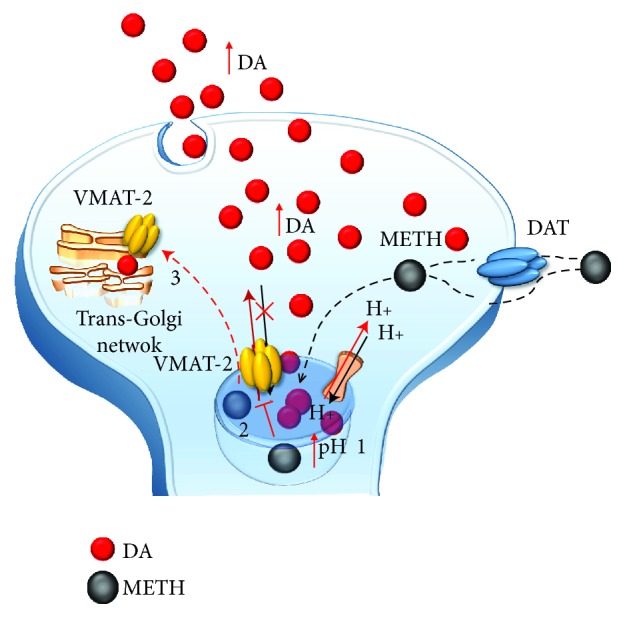
The effects of METH on DA-storing vesicles. METH enters into DA terminals either through the plasma membrane DAT or via passive diffusion. Within the axoplasm, it targets DA-storing vesicles to (1) disrupt their proton gradient, (2) inhibit and revert VMAT-2, and (3) displace VMAT-2 elsewhere (i.e., trans-Golgi network). These effects disrupt the physiological storage of DA, which diffuses from vesicles to the axoplasm and from the axoplasm to the extracellular space.

**Figure 2:**
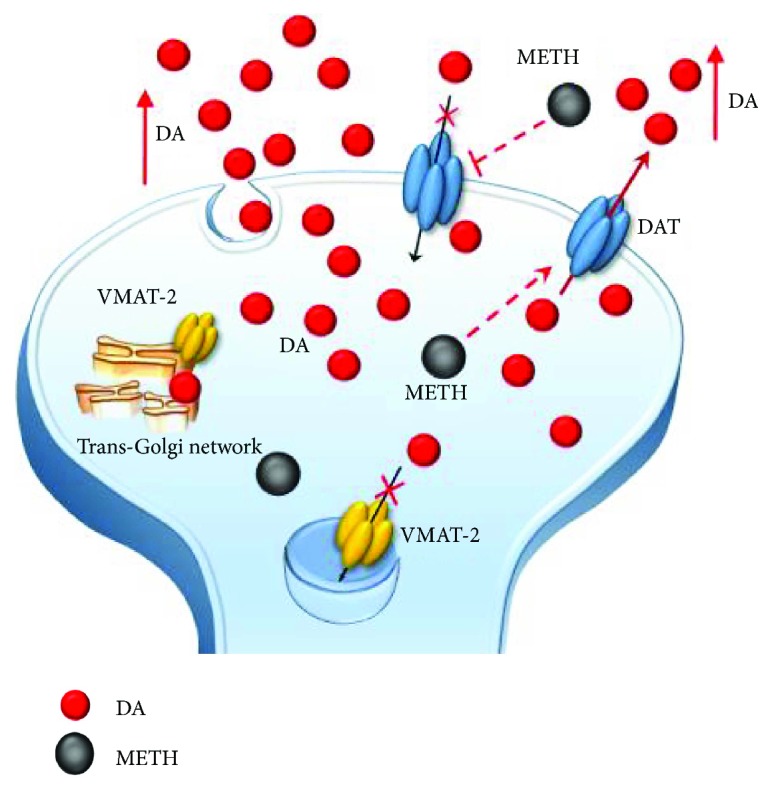
The effects of METH on DAT. METH impairs DAT activity either via direct inhibition or via reverting its direction. Such an effect potentiates the accumulation of freely diffusible DA in the extracellular space and prevents the main mechanisms of DA removal (reuptake within DA terminals).

**Figure 3:**
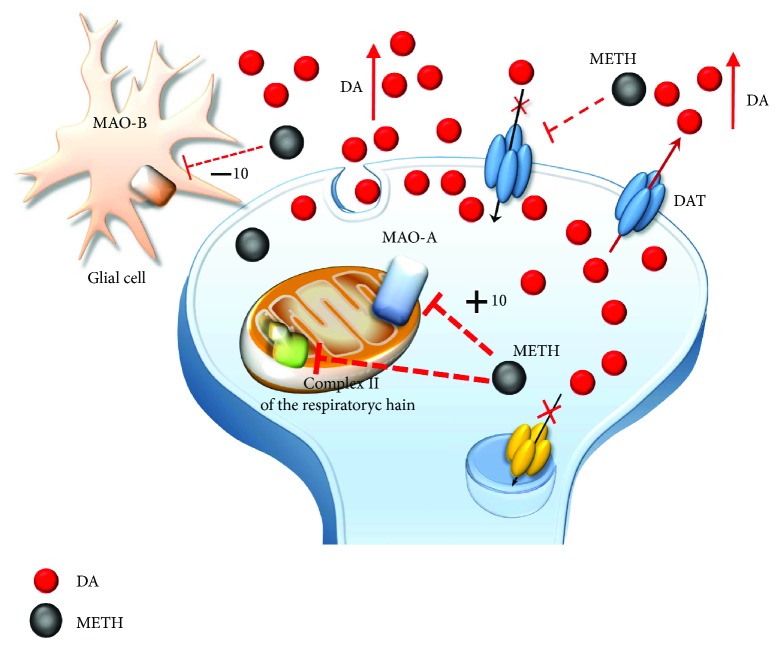
The effects of METH on mitochondria. METH impairs the activity of complex II of the mitochondrial respiratory chain and directly inhibits MAO-A placed on the outer mitochondrial membrane within DA terminals. METH also inhibits MAO-B placed extracellularly at the level of glia. However, the affinity of METH for MAO-B is tenfold less when compared with MAO-A. Thus, MAO-B inhibition does not influence that much the amount of extracellular DA.

Methamphetamine (METH) is a widely abused psychostimulant with powerful addictive and neurotoxic properties. This compound rapidly enters and persists within the central nervous system (CNS) [1, 2]. In fact, METH has a long half-life, which ranges from 10 to 12 hours [3]. METH kinetics within the ventral striatum parallel the time course of being “high” felt by METH users, who in fact, experience euphoria along with motor stimulation, excitation, increased energy, active waking state, sleeplessness, and alertness [4–6]. Such acute behavioral effects are due to early neurochemical events produced by METH, which consist in a rapid release of monoamines, mainly dopamine (DA), from nerve terminals. This occurs mostly within the striatum, where DA terminals are mostly abundant, though specific limbic regions and isocortical areas are involved as well [7–11]. The cellular effects induced by METH may be roughly summarized by its interaction with three molecular targets: (1) the synaptic vesicles and vesicular monoamine transporter type-2 (VMAT-2) (Figure 1). VMAT-2 belongs to the VMAT class of vesicular membrane proteins, which exist in two distinct forms, namely, VMAT1 and VMAT2. Both isoforms are responsible for the selective recognition and transport of cytosolic monoamines DA, norepinephrine (NE), and serotonin (5-hydroxytryptamine (5-HT)) within synaptic vesicles [12]. VMAT-2 and VMAT-1 are expressed in both neuronal and nonneuronal cells such as the chromaffin cells of the adrenal medulla. However, VMAT-2 prevails in the brain where it has a higher affinity for DA and NE compared with VMAT-1 [12]. VMAT-2 plays a key role in cytosolic DA homeostasis and release, since it guarantees the vesicular packaging and storage of both newly synthesized and synapse-recycled DA; (2) the plasma membrane DA transporter (DAT) (Figure 2), which selectively takes up extracellular DA within DA terminals; and (3) the monoamine oxidase (MAO) enzyme (Figure 3), which is the main intracellular enzyme responsible for the oxidative deamination of DA, NE, and 5-HT. MAOs exist as two different isoforms, MAO-A and MAO-B, which are placed at the level of the outer mitochondrial membrane of distinct vcell populations in the CNS [13]. In fact, MAO-A are present within catecholamine-containing neurons (DA, NE, and Epinephrine neurons), whereas MAO-B occur mainly in 5-HT cells and glia. Thus, the presence of MAO-A within DA terminals is crucial for the oxidative metabolism of intracellular DA, which together with VMAT-2 and DAT mediating DA uptake within the nerve terminals and within synaptic vesicles, respectively, represent the most powerful system to surveil DA activity. The activities of all these proteins are impaired by METH, once it enters the DA terminals via either passive diffusion or DAT.

**Figure 4:**
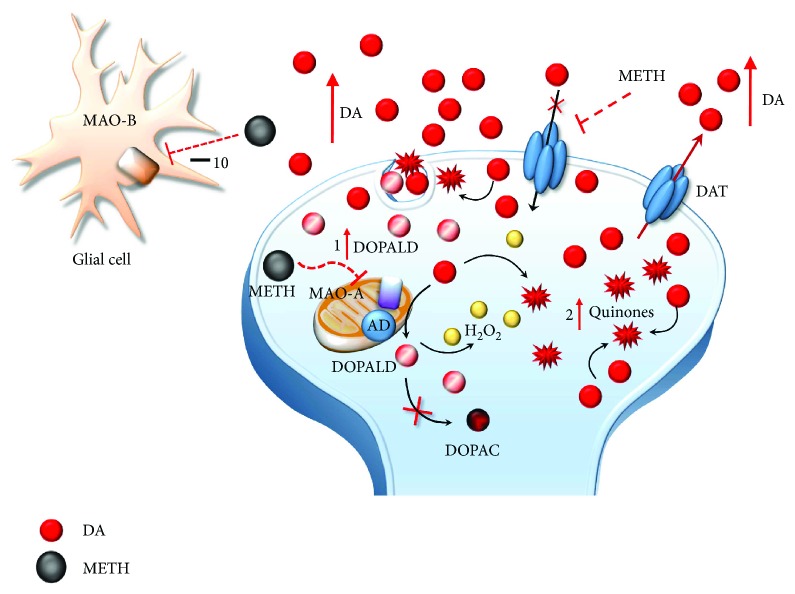
The effects of METH-induced MAO-A inhibition on DA metabolism. The loss of physiological DA deamination following MAO-A inhibition and its uncoupling with AD lead to the generation of highly reactive species including DOPALD (1), hydrogen peroxide (H_2_O_2_), and DA quinones (2).

In detail, at the level of synaptic vesicles, METH produces a variety of effects, which before affecting VMAT-2, are key in releasing DA (Figure 1). These effects are summarized as follows: (1) disruption of the proton gradient through the DA-storing vesicles due to the high pKa (pKa = 10.1) of METH, which rises the acidic compartment towards basic values, thus making nonpolar DA freely diffusible out of the vesicles [14–16]; (2) direct inhibition of VMAT-2 [17, 18], which prevents DA from reentering the vesicles; and (3) redistribution of VMAT-2 molecular complex from vesicle membranes to noncanonical membrane compartments such as those of the trans-Golgi network [19, 20], where DA may be inappropriately, though poorly, stored. The loss of physiological DA storage generates massive DA extravesicular levels within DA axons [21, 22] (Figure 1). It is noteworthy that a combined effect of METH as a weak base to tone down the pH gradient needs to be accompanied by a selective effect on VMAT-2 since alkalinization per se may be nonsufficient to fully produce the typical redistribution of vesicular DA [16]. This is confirmed by administering bafilomycin, which acts as a proton pump inhibitor only, with no effects on VMAT-2. Despite decreasing the pH ratio vesicle/cytoplasm 2-fold more than METH, bafilomycin redistributes only half of METH-induced DA levels in the extracellular compartment [23]. Once in the cytosol, METH also acts at the level of mitochondria (Figure 3) where two targets are affected: (4) METH inhibits complex II at the mitochondrial respiratory chain [24] and (5) METH inhibits MAO-A placed on the outer mitochondrial membrane [13]. This latter effect occurs as a competitive inhibition of METH upon MAO-A with a 10-fold higher affinity compared with MAO-B [13]. In both mice and humans, MAO-A are quite selectively placed within DA terminals. This is key since within DA terminals, MAO-A are coupled with aldehyde dehydrogenase (AD), which converts the highly reactive by-product of DA oxidation (3,4-dihydroxyphenylacetaldehyde (DOPALD)) into the quite inert 3,4-dihydroxyphenylacetic acid (DOPAC). The impairment of MAO-A may also include uncoupling between MAO-A and AD [25] (Figure 4).

**Figure 5:**
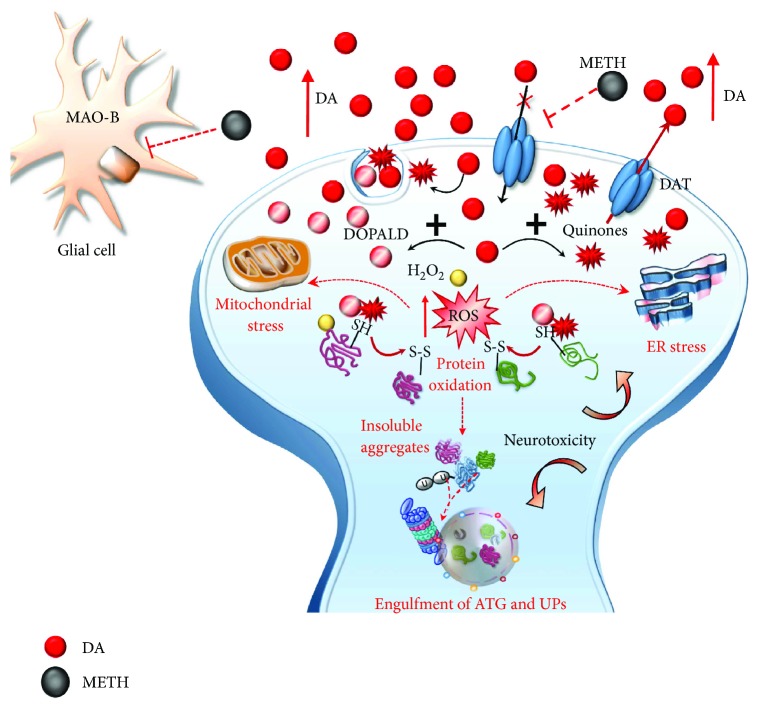
METH induces oxidative stress within DA terminals. Toxic DA by-products (quinones and DOPALD) together with highly reactive species such as H_2_O_2_ and reactive oxygen species (ROS) react with sulfhydryl groups and promote structural modifications of proteins within the DA axon terminals. The enhanced redox imbalance also disrupts the homeostasis of endoplasmic reticulum (ER) and mitochondria, which further accelerates the production of ROS. Thus, an excessive amount of misfolded/insoluble proteins and damaged organelles occurs, which leads to an engulfment of autophagy (ATG) and ubiquitin proteasome (UP) cell-clearing systems. These events converge in producing neurotoxicity within DA terminals, which may either extend to DA cell bodies.

In the absence of such a compartmentalized physiological oxidative deamination, DA autooxidation produces a high amount of reactive aldehyde DOPALD, which owns a dramatic oxidative potential and quickly interacts with surrounding proteins, by targeting oxidation-prone domains [25, 26]. Autooxidative DA metabolism leads to the generation of toxic quinones and highly reactive chemical species such as hydrogen peroxide (H_2_O_2_) and superoxide radicals, which in turn react with sulfhydryl groups and promote structural modifications of proteins, lipids, and nucleic acids within the DA axon terminals and surrounding compartments (Figure 5) [15, 27–39].

**Figure 6:**
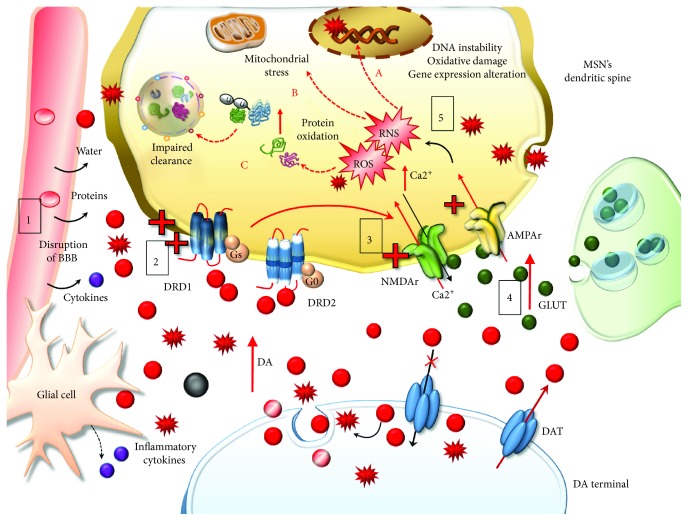
The effects of extracellular DA released following METH. Extracellular DA and DA-derived reactive species diffuse at considerable distance towards nonneuronal targets including the neurovascular unit (blood-brain barrier (BBB) and Glia), which is affected by METH (1). At short distance, METH produces an abnormal stimulation of postsynaptic neurons, mainly striatal MSNs. The pulsatile pattern of DA stimulation produces an abnormal pulsatile activation of postsynaptic DA D1 receptors (DRD1) (2). This leads to a series of noncanonical metabolic changes, which translate into activation of glutamate (GLUT) receptors N-methyl-d-aspartate and *α*-amino-3-hydroxy-5-methyl-4-isoxazolepropionic acid (NMDAr and AMPAr, resp.) (3) potentiation of GLUT release and Ca2+ entry within postsynaptic neurons (4). This event triggers an enzymatic cascade further increasing reactive oxygen species (ROS) and nitrogen species (RNS) (5). Freely diffusible DA-derived free radicals together with GLUT-derived radical species synergize to produce detrimental effects on postsynaptic non-DA neurons. These consist in DNA instability, due to oxidative damage (fragmentation and/strand breaks) and alterations in gene expression (A), mitochondrial stress (B), and oxidation of organic substrates, mainly proteins, which are prone to misfold and produce insoluble aggregates leading to an impairment of cell-clearing systems (C).

On the one hand, these effects drive a powerful oxidative stress for presynaptic DA terminals, which is key in producing nigrostriatal toxicity [27–31, 34, 40–43]. On the other hand, elevated cytosolic presynaptic DA diffuses in the extracellular space either by passive diffusion or via the reverted direction of DAT, another molecular effect which is promoted by METH (Figures 1–5) [14, 16, 33, 44]. All these effects also cause peaks of extracellular DA concentration, which produce synaptic effects at short distance. At striatal level, this paracrine environment encompasses medium-sized spiny neurons (MSNs). Nonetheless, due to the propensity of extracellular DA to diffuse at considerable distance from the DA terminals according to a volume transmission [45–47], other extrasynaptic sites may be affected as well. Such a paracrine spreading of extracellular DA is magnified during METH administration, since METH reverts DA uptake [33], thus preventing the main mechanisms of DA removal. This produces unusually high extracellular (and mostly striatal) DA levels which reach out nonneuronal targets including the neurovascular unit, which is also affected by METH administration [48, 49]. Intriguingly, the role of MAO-B enzymes in extracellular DA metabolism remains to be clearly established. In fact, although they occur outside DA cells, mainly within glia (Figures 3–5), they do not influence much the amount of extracellular DA [25, 50–53]. It is worth of noting that pulsatile METH intake/administration produces considerable oscillations of extracellular DA, which ranges from high peaks (exceeding 10-fold baseline levels) to severe deficiency (no detectable extracellular levels in brain dialysis techniques) within just a few hours [38, 54–56]. This pulsatile pattern of extracellular DA concentrations magnifies the slight variations produced by physiological release, such that, METH produces an abnormal stimulation (all and none) of postsynaptic neurons. For instance, pulsatile activation of postsynaptic DA receptors triggers noncanonical transduction pathways, which, along with the diffusion of abnormal reactive oxygen (ROS) and nitrogen (RNS) species, alter the response of postsynaptic neurons as mainly studied at the level of GABA MSNs [57–59] (Figure 6).

The impact of such a nonphysiological (in time, amount, and place) DA release is largely to blame when considering both the behavioral syndrome occurring immediately after METH intake and long-term behavioral changes including addiction, craving, relapse, and psychotic episodes, which reflect mainly the persistent alterations in postsynaptic DA brain regions following chronic METH exposure. As we shall see, overstimulation of postsynaptic DA receptors alternating with a lack of stimulation within an abnormal redox context drives most epigenetic effects. After mentioning the presynaptic effects of METH (to understand the role of redox species in causing the loss of integrity of DA axon terminals), the present review discusses the postsynaptic changes in relationship with epigenetics, DNA alterations, and persistent phenotypic changes produced by METH.

## 2. Presynaptic Effects of METH

In the present section, we wish to mention that METH produces presynaptic toxicity within DA terminals. As thoroughly revised by Moratalla et al. [34], the neurotoxic effects of high doses of METH, which occur both in a variety of experimental models and human abusers, are due to an excess of intracellular mostly DA-related, oxidative cascade (Figure 5). At first, such a toxicity was considered to be relevant only for DA axon terminals. Such neurotoxicity is documented by the following markers: (i) a steady decrease in striatal DA levels and striatal DA uptake sites [54, 60–63], (ii) loss of tyrosine hydroxylase (TH)-activity, TH immunoblotting and TH immunohistochemistry [64–71], and most directly, (iii) the occurrence of silver-stained (Fink-Heimer method) [61, 62] or amino-cupric silver-stained (de Olmos procedure) degenerating nerve fibers within the striatum [72]. Some studies also indicate the occurrence of METH-induced toxicity at the level of neuronal cell bodies in the substantia nigra pars compacta (SNpc). This was firstly reported by Sonsalla et al. [63], although this study was based on TH immunohistochemistry (which does not necessarily reflect an actual cell loss) and Nissl staining (with neither stereological counts nor positive evidence for a damage of the cell body). Further studies confirmed a loss of mesencephalic DA neurons even within the ventral tegmental area [67], but again, no stereological count was carried out. Other studies provided indirect evidence of cell death (TH immunohistochemistry, TUNEL assay, Fluoro-Jade B, or Nissl staining) [69–71, 73]. In a recent manuscript by Ares-Santos et al. [72], neuronal cell death of TH-positive neurons was visualized directly by using cupric silver staining (modified according to Beltramino and de Olmos). To our experience, a certain amount of cell loss is detectable only when very high doses of METH are administered, which corresponds to a loss of nigrostriatal DA terminals ranging over 80%, as demonstrated in the original article by Ares-Santos et al. [72]. Altogether, these findings are consistent with an increased risk to develop Parkinson's disease (PD), which is now quite well established in METH abusers [74–77]. Similarly to PD, METH produces neuronal inclusions in DA-containing PC12 cells and within SNpc neurons of mice [38, 78–84] as well as in humans [85]. These inclusions start as multilamellar whorls, which further develop as cytoplasmic inclusions reminiscent of PD-like Lewy bodies. In fact, these inclusions contain a high amount of ubiquitin and other proteins such as alpha-synuclein (*α*-syn), parkin, UchL1, and HSP70, which are typical markers of PD. Remarkably, most of these proteins are substrates of ubiquitin proteasome- (UP-) and autophagy- (ATG-) clearing systems, which are markedly affected during METH toxicity (Figure 5) [38, 79–81, 83, 86].

## 3. Postsynaptic Effects of METH

METH effects on postsynaptic compartment are multifaceted. Even neurotoxicity may extend to postsynaptic neuronal cell bodies throughout the striatum, hippocampus, and frontal cortex [72, 73, 87–94]. A pioneer manuscript by Jakel and Maragos [95] discussed very well how activation of DA receptors on striatal neurons as well as DA-derived oxidative species and oxyradicals might all converge to accelerate striatal neuronal cell loss in a specific striatal neurodegenerative disorder such as Huntington disease (HD). In fact, DA itself, DA-derived free radicals, and glutamate- (GLUT-) induced excitotoxicity may synergize to produce detrimental metabolic and oxidative effects on postsynaptic non-DA neurons (Figure 6). As we shall see, the interaction between DA and GLUT, as well as the convergence of signaling cascades placed downstream plasma membrane receptors, may be enhanced under chronic METH intake. In fact, striatal postsynaptic neurons increase their responsivity to both DA and GLUT following specific patterns of chronic METH administration. Along with diffusion of free radicals, which fuel oxidative damage, the striatal compartment challenged by METH is filled with DA acting on its receptors. It is well known that overstimulation of D1-like DA receptors (mainly D1 DA receptors (DRD1)) leads to a switch in the transduction pathway towards noncanonical signaling, which, in turn, generates a number of adaptive biochemical events [96–101]. This is evident when considering that an altered DRD1 signaling produced by METH enhances corticostriatal excitation by activating GLUT receptors and potentiating GLUT release. In extreme conditions, this may produce excitotoxicity within striatal GABA neurons [102–107]. In fact, increased extracellular GLUT and activation of its N-methyl-d-aspartate (NMDA) receptors promote calcium (Ca2+) entry within neurons as well as activation of nitric oxide synthase (NOS), which trigger an enzymatic cascade further increasing reactive oxygen species (ROS) and nitrogen species (RNS) [108–111]. Thus, following METH administration, GLUT synergizes with DA to produce oxidative stress, mitochondrial dysfunction, and inflammatory reactions, which synergistically interact to promote neuronal damage (Figure 6) [34, 42, 43, 109]. In line with this, cell inclusions filled with oxidized substrates are also detectable in the cytoplasm and within the nuclei of striatal GABAergic MSNs due to an overstimulation of DRD1 under METH administration [38]. This suggests that DA, acting on DRD1 joined with DA-derived free radicals, altogether may alter even nuclear signaling within GABA cells (Figure 6) [99]. Such an effect is expected to significantly alter DNA stability [29, 110–116].

Even the occurrence of striatal cytoplasmic inclusions within MSNs is likely to be due to combined mechanisms. In fact, it is well known that oxidative stress alters cell clearing systems, which is a seminal step in the generation of inclusion bodies containing oxidized/aggregated proteins [95, 117, 118]. At the same time, administration of DRD1 agonists reproduces neuronal inclusions within MSNs [38], which are prevented by DRD1 antagonists [38, 82]. Nonetheless, both DRD1 antagonists/deletion of the DRD1 gene and antioxidant compounds can protect from METH-induced oxidative stress and cell injury and retard/counteract behavioral sensitization in experimental models [34, 71, 96, 97, 119–125]. In summary, the various effects of DRD1 receptor overstimulation and prooxidative processes produced by excessive DA release are likely to assemble and cooperate to produce long-lasting neurochemical changes following METH.

## 4. Transcriptional and Epigenetic Effects of METH

A number of papers explored the mechanisms operating at postsynaptic level to modify neuronal phenotype, in an effort to unravel potential strategies to counteract addiction. To such an aim, in the last decades, a number of studies focused on specific transduction pathways and genes activated by METH. Remarkably, studies of the last decade indicated a key role for epigenetic mechanisms in modulating the transcription of a number of genes, which underlie long-lasting behavioral alterations and biochemical events induced by METH abuse. A gap still exists concerning the signaling cascades through which METH may induce epigenetic changes via mechanisms going beyond a mere effect of DA-related oxidative stress. In the present section, studies focused on METH-induced epigenetic changes in both experimental models and human abusers are discussed. In Sections 4.1 and 4.2, we focus on the effects of METH on the abnormal DRD1-mediated biochemical cascade, subsequent recruitment of specific second messengers and redox-sensitive transcription factors (TFs), and altered expression of immediate early genes (IEGs). At last, in Section 4.3, we touch on the evidence about how epigenetic remodeling may alter gene transcription thereby producing persistent behavioral changes, which define METH addiction.

### 4.1. DA D1-Like Receptor-Mediated Biochemical Events Induced by METH

**Figure 7:**
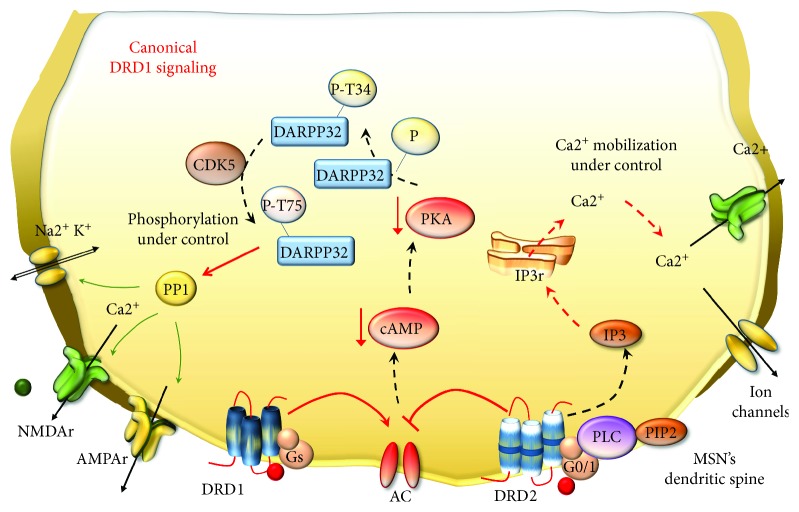
An overview of canonical DA receptor signaling. During physiologic DA stimulation, AC activity is balanced by the excitatory and inhibitory effects of DRD1 and DRD2, respectively. Thus, there is a physiologic downregulation of cAMP and PKA activation. PKA has a broad array of targets such as the DA- and cAMP-regulated phosphoprotein (DARPP-32), voltage-gated ion channels, and GLUT receptors. PKA phosphorylates DARPP-32 at Thr34 (P-T34), but other proteins, such as cyclin-dependent kinase 5 (CDK5), counterbalance such an effect by phosphorylating DARPP-32 at a different site (P-T75). Thus, DARPP-32 can activate phosphatase protein 1 (PP1), which can surveil phosphorylation levels of all PKA targets. Likewise, canonical stimulation of DRD2, which are coupled with PLC, generates normal levels of inositol 1,4,5 trisphosphate (IP3) which induces Ca2+ release from the endoplasmic reticulum. Since ion channels and GLUT receptors are properly functioning, intracellular Ca2+ can be efficiently mobilized.

DA canonical signaling in the brain is mediated by five (DRD1–DRD5) G-protein-coupled receptors, which are grouped into two classes depending on which G-protein they are coupled to. D1-like receptors include DRD1 and DRD5 and they stimulate G_s_ and G_olf_ proteins, which activate adenylate cyclase (AC), thus elevating intracellular levels of cyclic adenosine monophosphate (cAMP) to increase protein kinase A (PKA) [126]. On the other hand, D2-like receptors (DRD2, DRD3, and DRD4) stimulate G_o/i_ proteins [126] and they act by inhibiting AC [127]. Again, these receptors target voltage-dependent ion channels through a mechanism, which operates at the level of plasma membrane and phospholipase C (PLC) [128]. All five DA receptors are expressed in the striatum, but DRD1 and DRD2 are the most abundant, with the former being placed specifically within postsynaptic neurons and the latter being placed both presynaptically and postsynaptically (Figure 7).

**Figure 8:**
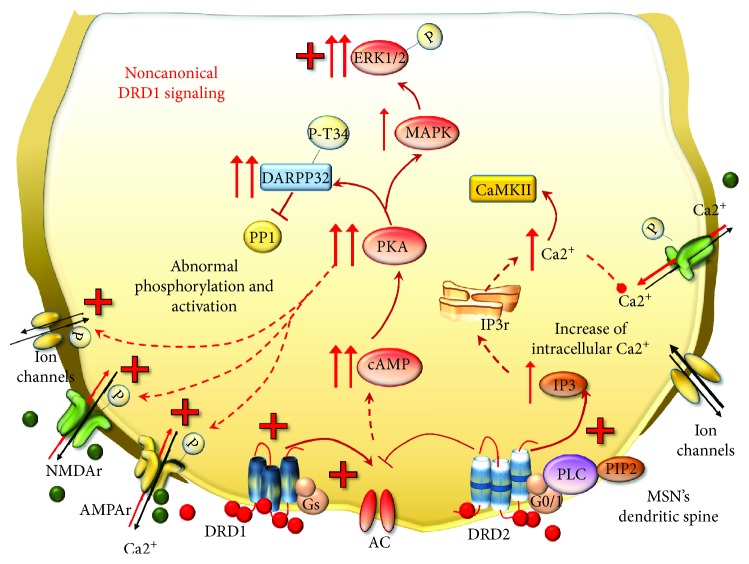
METH-induced noncanonical DRD1 signaling. Following METH, MSNs become supersensitive to pulsatile DA stimulation despite that the number of DA receptors is not increased. As a result, DRD1 move towards noncanonical signaling and the activity of DRD2 is enhanced. In these conditions, DRD1 overactivates AC, which enhances the production of cAMP and leads to abnormal activation of PKA. DRD1/PKA cascade turns out to increase the amount of DARPP-32 phosphorylated at Thr34, which inhibits PP1. Thus, all PKA targets, including voltage-gated ion channels and GLUT NMDAr and AMPAr, are abnormally phosphorylated and activated. In addition, DRD1/PKA leads to increased levels of MAPK and ERK1/2, which in turn phosphorylate several cytosolic and nuclear substrates. At the same time, DRD2-enhanced activity potentiates the increase of intracellular Ca2+ release, which cannot be properly mobilized, since ion channels and GLUT receptors are abnormally activated and potentiate the influx of Ca2+ within postsynaptic neurons. Such an event also promotes the activation of calmodulin-dependent kinase II (CaMKII), which can translocate into the nucleus to regulate gene expression.

For such a reason, DRD1 and DRD2 represent a cue investigation topic in the context of behavioral effects underlying drug addiction. However, the disruption of canonical DRD1 signaling is more important [57, 99, 129]. In fact, peaks and drops of DA stimulation generate the switch from canonical to noncanonical DRD1 signaling. This occurs during METH abuse in a way that is reminiscent of DA replacement therapy in advanced PD [57, 99, 130–139]. This kind of perturbation of DRD1 is the authentic drive to switch the DRD1 transduction pathway [59, 99, 131, 137]. Thus, in the presence of abnormal stimulation, DRD1 moves towards noncanonical signaling which makes MSNs supersensitive to DA stimulation despite that the number of DA receptors is not increased [99]. In fact, a chain of events follows DRD1 overstimulation, which involves metabolic transduction and transcriptional pathways, eventually switching gene expression and neuronal phenotype underlying addictive behavior in PD and METH [57, 59, 96–99, 119, 121, 132, 140–144]. Although precise signaling changes and substrates underlying this shift remain to be fully elucidated, a prominent role for AC [145] and PKA [146, 147] is well established (Figure 8). In fact, in its canonical pattern, PKA phosphorylates cellular targets, including voltage-dependent ion channels, GLUT receptors, TFs, and epigenetic enzymes involved in physiological synaptic plasticity and synaptic strength as naturally occurring in a normal striatum. When the noncanonical DRD1 transduction pathway is activated, PKA recruits mitogen-activated protein kinases (MAPKs) and extracellular signal-regulated kinases 1/2 (ERK1/2) [132, 148–153]. ERK1/2 proteins may translocate into the nucleus to phosphorylate and activate several TFs, such as the cAMP response element-binding protein (CREB), Elk-1, nuclear receptors, and H3 histones, which all regulate gene expression [152, 154–156]. A key substrate of DRD1/PKA signaling in the striatum is the DA- and cAMP-regulated phosphoprotein (DARPP-32). Following persistent METH-induced pulsatile DRD1 overstimulation, DARPP-32 is chronically enriched and abnormally phosphorylated in MSNs, where it serves neuroplastic changes and behavioral sensitization [155, 157–161]. In fact, DRD1-activated PKA directly phosphorylates DARPP-32 at threonine 34 (Thr34), which accumulates in the nucleus, where it may promote directly histone phosphorylation [162–164]. Moreover, phosphorylation at Thr34 induced by PKA converts DARPP-32 into a powerful inhibitor of protein phosphatase1 (PP1) [163], leading to abnormal PKA-mediated phosphorylation [129]. This eventually alters the very same substrates known to be affected by METH including (i) ion and voltage-gated channels and pumps, such as Ca2+ channels, Na^+^ channels, Na^+^, and K^+^ ATPases [58, 59, 165]; (ii) GLUT receptors including *α*-amino-3-hydroxy-5-methyl-4-isoxazolepropionic acid (AMPA) and NMDA and their subunits GluR1 and NR1, respectively [166–173]; (iii) GLUT transporters VGLUT-1 and EAAT3 [174, 175]; (iv) GABA receptor subunits [176]; and (v) TFs, such as CREB [168] (Figure 8).

**Figure 9:**
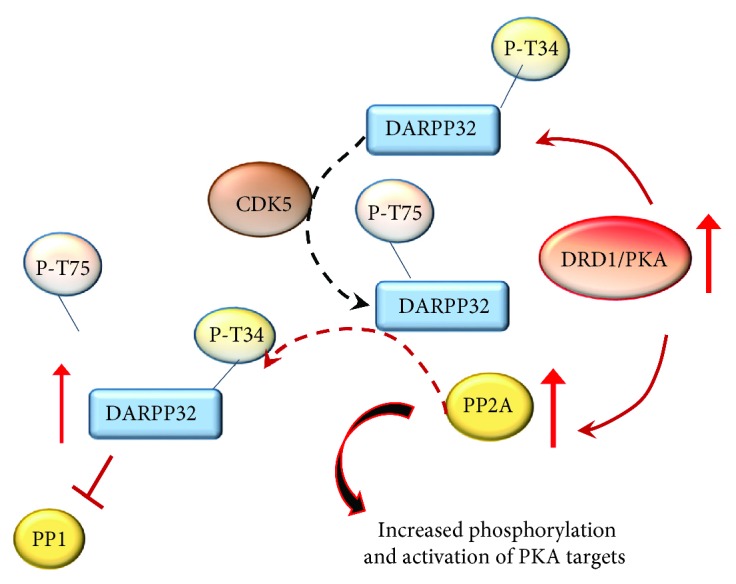
The effects of DRD1/PKA pathway on CDK5 and DARPP-32. In physiologic conditions, CDK5 phosphorylates DARPP-32 at Th75, thus softening the effects of PKA on DARPP-32. However, the abnormal phosphorylation of Thr34 carried out by enhanced DRD1/PKA cannot be counterbalanced by CDK5. This occurs since DRD1/PKA activates phosphatase PP2A, which inhibits the effects of CDK5 and enhances those of PKA. As a result, DARPP-32 phosphorylated at Thr34 increases and potentiates the inhibition of PP1.

TFs, in turn, may either induce or suppress a number of downstream target genes. Noteworthy, noncanonical DRD1 stimulation initiates a vicious circle of reciprocal enhancement between DRD1 and GLUT receptor activities, since once activated, NMDA and AMPA receptors promote themselves a noncanonical phosphorylation of DARPP-32 and CREB in the striatum [168, 177]. These findings are in line with evidence showing that, following amphetamines, NMDAr and DRD1 synergistically activate ERK signaling within MSNs of the dorsal striatum and nucleus accumbens (NAc) [155]. Remarkably, regulation by DARPP-32 occurs both upstream of ERK and at the level of the downstream-activated striatal-enriched tyrosine phosphatase (STEP), which demonstrates its cyclic functional relevance [155]. In summary, ERK plays a primary role in mediating long-lasting effects of psychostimulants within the striatum (especially dorsal striatum and NAc). In fact, blockade of the ERK pathway or mutation of DARPP-32 alters locomotor sensitization induced by amphetamines [155]. Altered signaling during DRD1 overstimulation also applies to cyclin-dependent kinase 5 (CDK5), which is recruited by both DRD1 and NMDAr in the striatum [178]. In physiological conditions, CDK5 phosphorylates DARPP-32 at threonine 75 (Thr75), thus inhibiting the phosphorylation of Thr34 carried out by PKA [157, 179, 180]. The decreased phosphorylation of DARPP-32 at Thr34 could decrease PKA activity; however, in the context of noncanonical signaling, there is an activation of phosphatase protein PP2A, which in turn dephosphorylates DARPP-32 at Thr75. In this way, PKA activity turns out to inhibit CDK5-DARPP-32/Thr75 activity [163, 181]. Such a switch is typical of noncanonical DRD1 signaling triggered by DRD1/PKA pulsatile activation. Therefore, in the presence of DRD1 overstimulation, a sustained CDK5-mediated mechanism would fuel, rather than dampen, the phosphorylation of DARPP-32 at Thr34 (Figure 9).

In this way, the cyclic signaling pathway of CDK5 and DARPP-32 in the striatum represents an endogenous feedback mechanism, which is likely to enhance the phosphorylation of various substrates thus sustaining the sensitized behaviors produced by reiteration of pulsatile DRD1/PKA stimulation. In line with this, the activity of CDK5 is implicated in motor- and reward-related behaviors following drug abuse including METH [160, 178, 182, 183].

In addition to the mechanism described above, DRD1 signaling may also activate PLC to generate inositol 1,4,5 trisphosphate (IP3) which participates in Ca2+-regulated signaling pathways [184–187]. In fact, DA was reported to generate robust intracellular Ca2+ oscillations in about 40% of striatal MSNs via a DRD1-dependent mechanism involving both PKA and PLC [184]. Nonetheless, recent studies indicate the existence of DRD1-DRD2 heterodimers that require a coincident activation of both receptors for intracellular Ca2+ release. This is coupled with activation of a calmodulin-dependent kinase II (CaMKII), which translocates in the nucleus to regulate gene expression [185–187]. Taken together, these observations suggest that multiple interactions exist between PKA, PLC, and intracellular Ca^2+^ transduction mechanisms within DRD1-expressing striatal MSNs. In response to neurotransmitter receptor activation and enhanced oxidative stress, specific TFs are recruited to regulate gene transcription. These TFs are often present within large protein complexes, which bind to a specific DNA sequence corresponding to promoter or enhancer regions of target genes. In the next paragraph, we will focus on those TFs and genes recruited during METH administration according to the biochemical pathways we just described.

### 4.2. Transcription Factors and Immediate Early Genes (IEGs) Induced by METH

**Figure 10:**
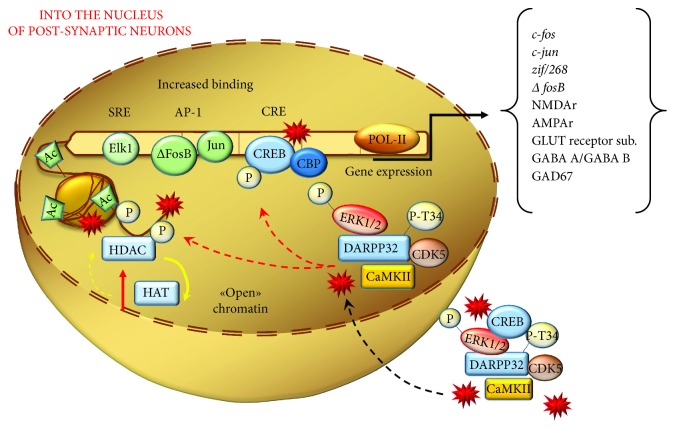
The nuclear effects of DRD1/PKA pathway and reactive species on postsynaptic neurons. The noncanonical DRD1 activation induced by METH produces an overactivation of several kinases, such as ERK1/2, DARPP-32-pT34, CREB, CDK5, and CaMKII. The latter, together with DA- and GLUT-derived reactive species, is shuttled into the nuclear compartment where they carry posttranslational modifications of histones and TFs. These events promote both a relaxation of chromatin structure (yielded by an increase of histone acetyltransferases (HAT)/decrease of histone deacetylases (HDAC)) and increased binding of TFs (such as Elk-1, AP-1, and CREB) at the level of their target gene sequences. These metabolic events eventually translate into an increase expression of IEGs.

The cascade of biochemical events mediated by the combined effects of DA and oxidative species following METH administration activates a plethora of TF families beyond CREB, encompassing activator protein 1 (AP-1), early growth response (Egr) proteins, Elk-1, nuclear factor of activated T-cells (NFAT), nuclear factor *κ*B (NF*κ*B), which modulate the expression of several IEGs [98, 100, 110, 122, 123, 125, 154, 188–198]. By definition, IEGs undergo early synthesis and they can associate to form a variety of homo- and heterodimers binding to common DNA sites to regulate further gene expression. This leads to a variety of plastic effects ranging from neuronal metabolism to neuromorphology. In line with this, METH alters the expression of a multigene machinery coding for proteins involved in signal transduction, metabolic pathways, and transcriptional regulation. This alters protein expression and alters the amount of inflammatory cytokines, neuropeptides, and trophic factors (mainly brain-derived neurotrophic factor (BDNF)), as well as oxidative-, mitochondrial-, and endoplasmic reticulum stress-related events and proapoptotic cascades [91, 122, 125, 197–210]. DA-related events and oxidative mechanisms converge to alter TF expression following METH (Figure 10).

On the one hand, DA per se and its metabolites provide a powerful source of radical species, which in turn interact with DNA and TFs to modulate gene expression [110, 115, 211]. In fact, ROS can alter the DNA-binding activity of diverse TFs, by oxidizing DNA bases or specific amino acidic domains (mainly cysteine and lysine residues) of histones and/or TFs. ROS also act as signaling molecules and second messengers by activating intracellular cascades such as MAPKs. These effects converge in recruiting TFs such as AP-1 and NF*κ*B [110, 115, 211], which govern the expression of specific IEG coding for proteins involved in neuronal functions such as death and survival control, cellular defense mechanisms, and immunological and inflammatory responses, which in turn are a powerful source of ROS. On the other hand, several studies have shown that genetic or pharmacological repression of DRD1 can revert METH-induced activation of redox-sensitive TFs, including AP-1, NF*κ*B, CREB, Egr, and NFAT by producing a normalization of the levels of IEGs [122, 123, 212–215].

For instance, CREB, which during baseline DA stimulation is slightly recruited, becomes overactive in the presence of pulsatile DA levels, which lead to a DRD1/PKA-mediated aberrant phosphorylation cascade driven by oxidative stress and/or DRD1 overstimulation [129, 168, 216]. CREB activates genes through the binding to cAMP-responsive element (CRE). Phosphorylation of CREB by PKA at serine119 is required for its interaction with DNA, while phosphorylation at serine-133 allows CREB to interact with CREB-binding protein (CBP) in the nucleus. Members of the CREB gene family include activating transcription factors 1–4 (ATF 1–4), CREB-1, and CREB-2, and each of the ATF/CREB proteins can bind to CRE motifs as either homodimers or heterodimers. In fact, METH administration increases both phosphorylated CREB (pCREB) and members belonging to the CREB family, which then bind to CRE motif of several genes to increase their expression [91, 122, 190, 197, 205, 209, 210, 215, 217–220]. In addition, ATF/CREB dimers also bind to Fos/Jun members belonging to the AP-1 family, thus forming cross-family heterodimers [221]. AP-1 is mainly known for its role in cell proliferation, while it plays a compensatory effect on redox stress and DNA damage [222]. AP-1 DNA-binding complex is in fact a dimer composed of IEGs, which are members of Jun (c-Jun, Jun B, and Jun D) and Fos (c-Fos, Fos B, Fra-1, and Fra-2) TF families [222]. Several studies demonstrate that METH causes an early increase in IEG expression belonging to Jun and Fos families [91, 123, 197–199, 205–207, 209, 212, 218, 219, 223–227]. Among these genes, a special emphasis is given to ΔFosB, which consists of a stable splice variant of FosB. In fact, differently from other Fos/Jun family proteins featuring a transient induction by acute drug exposure, the increase in ΔFosB (mRNA and/or protein) persists for longer time intervals within the striatum [228, 229]. In line with this, ΔFosB may play a key role in triggering addiction [209, 218, 228, 230–232]. This posed ΔFosB as a master regulator of persistent nuclear effects induced by METH, which are the core of METH-related epigenetics. Thus, reaching out ΔFosB is considered as a key point to trigger persistent epigenetic changes through persistent alterations of transcriptional regulatory proteins (including CDK5 and epigenetic enzymes), which all influence the phenotype of MSNs [229].

**Figure 11:**
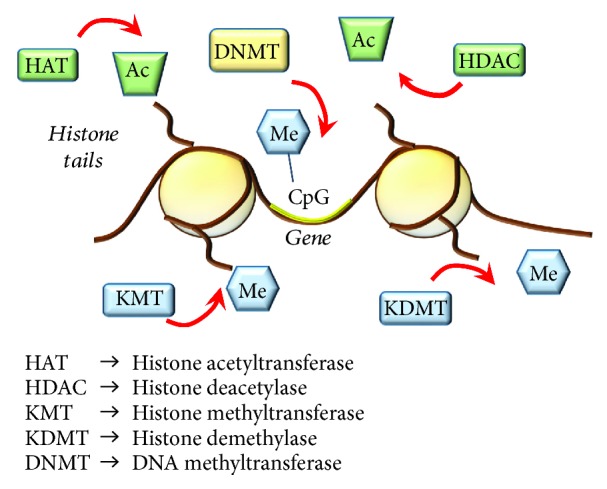
Summarizing main epigenetic mechanisms. This cartoon roughly reports the main epigenetic enzymes carrying structural modifications of lysine (K) residues of histone tails and DNA promoter sequences at the level of CpG islands. HATs act by adding acetyl groups (Ac) which associates with increased gene expression; HDACs repress gene expression by removing Ac from K histone residues; KMT transfer methyl (Me) groups and KDMT remove Me groups from K histone residues; the effects of KMTs and KDMT on gene transcription depend on the specific histone K that is modified; DNMTs mediate increased methylation of cytosine (C) residues in CpG islands of gene promoters, which associates with repressed gene expression.

ΔFosB-related epigenetic changes occurring in various nuclear sites mainly consist in acetylation/deacetylation and methylation/demethylation at the level of histones or DNA (Figure 11).

Those simple phenomena occurring in specific sites and critical time windows generate the remarkable diversity and specificity in the epigenetics of METH. In fact, the transcriptome/exome alterations generated by METH-induced epigenetics create the specific structural plasticity that we appreciate within MSNs. This is achieved by diverse effects on a number of genes. A critical site concerns genes involved in building the architecture of dendritic spines, such as GLUT NMDAr [108] and AMPAr [233] subunits, GABA-A [176] and GABA-B [233] receptor subunits, and the GABA-synthesizing enzyme GAD-67 [176] (Figure 10).

Beyond ΔFosB, the Egr family represents another subclass of zinc finger structural motifs involved in eukaryotic protein-nucleic acid interaction. Members of Egr include IEGs such as Egr1 (Krox-1, NGF1A, and Zif268), Egr2 (Krox20, NGF1B), Egr3 (Pilot), and Egr4 (NGF1C), which are regulated by posttranslational changes such as phosphorylation and redox state [234]. In line with this, METH activates and overexpresses several members of the Egr family, especially Egr1 and Egr2 [91, 123, 188, 198, 212, 225, 235–237].

Again, METH causes substantial increases in the expression of nuclear TF families including nuclear receptor 4a (Nr4a), nuclear factor erythroid 2- (NFE2-) related factor 2 (Nrf2), and NFAT, which regulate genes involved in metabolism, development, and axonal growth within the mammalian brain [91, 225, 226, 237]. In detail, METH produces a shuttling of NFATc3 and NFATc4 from the cytosol to the nucleus [91, 195]. Similar findings were reported for the DNA-binding protein NF*κ*B, which, following METH, redistributes to the nucleus of striatal neurons [197, 204, 208, 209, 242]. NF*κ*B is rapidly activated and overexpressed by METH. In detail, once in the nucleus, NF*κ*B promotes a vicious cycle of oxidative events, which include an increased expression of inducible nitric oxide synthase (iNOS) and cyclooxygenase-2 (COX-2) to generate nitric oxide (NO), prostaglandins, and inflammatory cytokines as well as activation of the apoptosis-promoting factor p53 [200, 201, 238].

**Figure 12:**
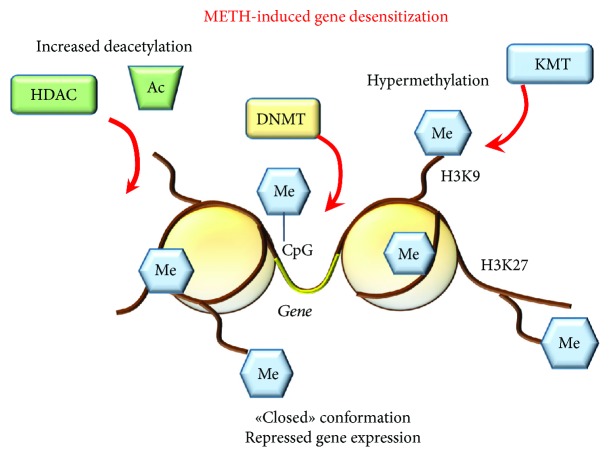
METH-induced gene desensitization. Exposure to chronic METH produces epigenetic effects, which repress further gene expression. This occurs mainly through increased activity of deacetylation enzymes (HDAC), increased methylation of lysine 9 and 27 (K9/K27) residues of histones (i.e., H3K9/27) by methyltransferases (KMTs) and hypermethylation of gene promoters by DNA methyltransferases (DNMTs), which produce a “closed chromatin” conformation. Me: methyl groups; Ac: acetyl groups.

A critical point to decipher the effects of METH upon the activity of all these TFs is the pattern of drug administration. Again, early time intervals compared with late time intervals from METH exposure (i.e., withdrawal time) make a substantial difference. In most cases, acute METH induces an early activation of TFs, which is followed by upregulation of most IEGs. This early effect is short lived, which makes it unlikely to produce behavioral sensitization. This is confirmed by the fact that chronic METH administration produces opposite changes mainly featuring a downregulation of IEGs. Remarkably, chronic METH also blunts the effects of an acute single METH injection on several striatal IEG expression [237] which is more reminiscent of a “gene desensitization” (Figure 12).

**Figure 13:**
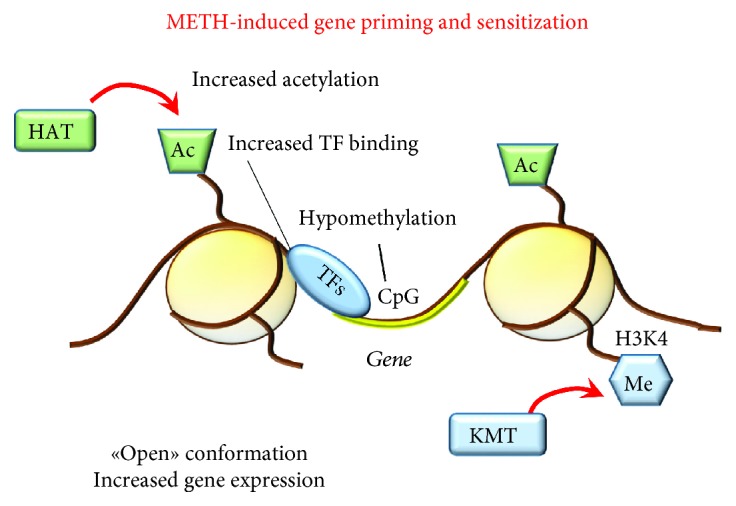
METH-induced gene priming and sensitization. A single dose of METH may be sufficient to induce an epigenetic switch consisting in increased gene expression. Such an effect may also occur during chronic METH resulting in long-term sensitization. This occurs through increased histone acetylation and methylation at specific K residues (i.e., H3K4) joined with poor activity of DNMTs (hypomethylation of CpGs), which altogether produce an “open” chromatin conformation and allow the binding of TFs at the level of gene promoters.

Conversely, a single exposure to a subthreshold dose of METH may suffice per se to induce a persistent increased response to further administration [239], a phenomenon which mirrors the “gene priming” (Figure 13). In fact, the effects of a single dose of METH on specific genes are markedly different depending on the existence of a previous METH exposure [240, 241]. These differences appear to be related to the occurrence of a previous epigenetic switch [229, 242].

### 4.3. METH as a Brain Epigenetic Modifier

Epigenetics in the CNS is currently accepted as the set of mitotic changes in gene transcription and/or phenotypic alterations that occur in the absence of modifications to DNA sequence itself [243]. Dynamic epigenetic remodeling allows perpetual alterations in gene readout within cells, and within the CNS, it may have a crucial impact on neuronal function. Posttranslational modifications of histone proteins, changes in the binding of TFs at gene promoters, and covalent modifications of DNA bases represent the main mechanisms through which gene expression is regulated. Over the past decade, studies investigating the regulation of transcription, through modifications of DNA (hypo-/hyper-/hydroxymethylation of cytosine residues) and chromatin structure (acetylation and methylation of histones) (Figure 11), have exploded in addiction research [244–246]. In recent years, METH was shown to induce epigenetic modifications, which underlie persistent changes in gene expression and long-lasting behavioral responses to the drug [198, 210, 228, 237, 247–251].

#### 4.3.1. METH and Histone Acetylation

Histone acetylation and deacetylation are a dynamic process balanced by histone acetyltransferase (HAT) and histone deacetylase (HDAC), a subset of enzymes, which carry out reversible histone modifications by adding or removing acetyl groups. In general, by adding acetyl groups to histones, HATs promote gene expression by creating an “open” chromatin conformation, while HDACs produce a “closed” conformation and represses transcription by removing acetyl groups [252]. These enzymes physically interact with sequence-specific TFs and target-specific promoters, to modify acetylation patterns of core histones, thus manipulating the functional state of chromatin and orchestrating the transcriptional machinery [252]. The HAT families include CREB-binding protein (CBP)/p300, while HDACs can be classified into four families according to sequence similarities [253]. These include class I (HDAC1, HDAC2, HDAC3, and HDAC8), class II (HDAC4, HDAC5, HDAC6, HDAC7, HDAC9, and HDAC10), class III (sirtuins, SIRTs 1–7), and class IV (HDAC11) HDACs. HDACs are widely implicated in synaptic plasticity and long-term memory, which is key in drug addiction [254].

*(1) Histone Acetylation and Increased Gene Expression*. *Acetylation-Related Transcriptional Effects of Acute METH.* Several studies documented that METH at short-time intervals increases H4 acetylation (H4K5ac and H4K8ac) in the rat NAc and striatum [225, 228, 255, 256]. This associates with increased gene expression detected at early time intervals following METH [225, 255]. In detail, such an increase (mainly concerning IEGs such as Egr1, Egr2, c-Fos, JunB, Nr4a3, and corticotrophin releasing factor (Crf)) correlates with increased binding of H4K5ac to the promoters of these very same genes [225, 228, 255]. METH-induced H4 acetylation may follow either decrease in HDAC1 expression or increase in CBP expression in the Nac [255]. In fact, acute METH also induces an increase of ATF2, a member of the ATF/CREB family [255], which behaves as a HAT by acetylating histone H4 [257].

*Acetylation-Related Transcriptional Effects of Chronic METH.* In 2013, Krasnova et al. [210] used an experimental model of chronic METH self-administration in order to decipher large-scale epigenetic and transcriptional changes occurring specifically within the NAc and dorsal striatum, to explain compulsive behavior characterizing drug addiction [258]. In detail, METH self-administration enriches pCREB on the promoters of genes coding for c-Fos, FosB, BDNF, and (synaptophysin) Syp. Both pCREB and gene expression followed the same expression pattern being upregulated at 2 h after drug intake and going back to normal levels at 1 month of withdrawal. This suggests that CREB is relevant as an epigenetic mediator of transcriptional changes produced by METH. In contrast to c-Fos mRNA, chronic METH self-administration does not affect c-Fos protein levels after 2 or 24 h. Remarkably, at 1 month of METH withdrawal, c-Fos protein was found to be decreased compared with controls. In contrast, no changes were observed in ΔFosB mRNA levels, while ΔFosB protein was significantly increased at 2 and 24 h after chronic METH self-administration. Similarly to c-Fos, ΔFosB decreased at 1 month of withdrawal [210], which dampens an exclusive role of ΔFosB as an irreversible switch for addiction.

*(2) Histone Deacetylation and Decreased Gene Expression*. *Deacetylation-Related Epigenetic Effects of Acute METH.* A recent study shows that in HDAC2-KO mice, METH produces a greater increase in some IEG transcripts (FosB, Fra-2, Egr1, and Egr3) when measured at early time interval (1 h postinjection) [227]. The levels of these transcripts persist for 2 hours in HDAC2-KO mice. In contrast, in WT mice, at 2 h, these IEGs are suppressed. This demonstrates that METH recruits HDAC2 to the promoters of these IEGs thereby bringing back transcript levels to normal values. Remarkably, in HDAC2-KO mice, the persistency of IEG expression is correlated with increased enrichment of pCREB on the promoters of the very same genes. Downregulation of other genes (follistatin (Fst), inhibin beta A (InhbA), neuromedin U (Nmu), cholecystokinin (Cck), and BDNF), which occurs at delayed time intervals (8, 12, and 24 h) after METH administration, combines with increased expression of HDACs in the Nac and dorsal striatum [255]. In fact, METH decreases histone H3 acetylated at lysine 9 and 18 (H3K9Ac and H3K18Ac) on the promoters of these genes [255].

*Deacetylation-Related Epigenetic Effects of Chronic METH*. Renthal et al. [228] found that, at 5 days of amphetamine withdrawal, when c-Fos was maximally repressed, ΔFosB accumulated on c-Fos promoter, suggesting that ΔFosB desensitizes c-Fos expression. Conversely, the HDAC inhibitor sodium butyrate reverts METH-induced repression of c-Fos, supporting the idea that hypoacetylation on the c-Fos promoter desensitizes the gene [228]. The question of whether ΔFosB remains steady linked to specific gene promoters for longer periods of time or ΔFosB alters gene inducibility by producing long-lasting chromatin changes still remains to be elucidated.

McCoy et al. [237] showed that chronic METH reduces the expression of several TFs and IEGs (i.e., AP1, Erg1-3, and Nr4a1) way below control levels. This occurs along with decreased CREB expression. Remarkably, chronic pretreatment with METH suppresses the stimulatory effects on these IEGs when elicited by an acute challenge with the drug. This paradoxical response occurs along with a greater decrease in CREB levels compared with those measured during chronic administration [237]. Similar findings were produced by Cadet et al. [225], who reported that a challenge of METH to rats treated chronically leads to downregulation of 53 out of 71 genes. These effects were related to decrease H4K5Ac binding. In addition, chronic administration of low METH doses decreases the abundance of H4K5ac, H4K12ac, and H4K16ac on the promoters of genes coding for GluA1-2 and GluN1 subunits of AMPAr and NMDAr, respectively [259]. Accordingly, there was a decrease in the expression of GLUT receptors causing a decrease in the current generated by GLUT stimulation. These phenotype changes occurred along with increased striatal expression of HDAC1, HDAC2, SIRT1, and SIRT2. A causal relationship is strengthened by the opposite effects produced by the HDAC inhibitor valproate, which prevents METH-induced alterations at the very same receptor subunits [259]. When METH is administered in higher doses, a change in the expression of different classes of HDACs is found [260]. This confirms a dose dependency for METH-induced epigenetic alterations. In fact, depending on the dose of METH being administered, sometimes, opposite phenotypic changes occur. METH was shown to upregulate other epigenetic proteins including methyl CpG-binding protein 2 (MeCP2), repressor element-1 silencing transcription factor (REST), and corepressor-REST (Co-REST), which are members of corepressor complexes with class I HDACs [259]. Among these, the multifunctional complex MeCP2 received some attention since METH increases MeCP2 expression in the ventral and dorsal striatum [261, 262].

#### 4.3.2. METH and Histone Methylation

Histone methylation is regulated by enzymes that add methyl groups acting as writers, namely, methyltransferases (KMTs) and enzymes that remove methyl groups, acting as erasers, namely, demethylases (KDMTs). KMTs are involved in mono-, di-, and trimethylation of histone lysine residues (K), which carry specific regulatory switches [263]. In fact, histone methylation regulates both repression and activation of gene expression, depending on the specific K being modified. For instance, methylation of histone H3 at K4 (H3K4me) is associated with increased transcriptional activity whereas methylation of H3 at K9 (H3K9me) and K27 (H3K27me) is associated with repression of gene expression [263]. Moreover, several classes of KDMTs may counteract the effects of the KMTs by erasing methyl moieties.

Several studies demonstrated the involvement of KMTs and KDMTs in METH addiction [228, 247, 248]. For instance, the study of Renthal et al. [228] demonstrated that in addition to the role of HDAC1, repression of c-Fos at 5 days after drug withdrawal was associated with amphetamine-induced increase of H3K9me2 on the promoter of c-Fos. This effect correlates with increased expression levels of KMT1A. More recently, epigenetic mechanisms contributing to METH-associated memories were explored in the NAc and dorsal striatum, given their role as a hub for drug craving. While investigating such a phenomenon, Aguilar-Valles et al. [247] provided evidence that genetic ablation of KDM5C demethylase increases H3K4me at the level of promoters of IEGs including Fos and oxytocin receptor gene (Oxtr), which associates with increased METH-associated memory. On the contrary, KO mice for MLL1 (mixed lineage leukemia, a member of the KMT family) which possess decreased H3K4me and transcript levels of Fos and Oxtr genes show reduced METH-associated memory [247]. Again, METH craving was shown to be related with epigenetic changes occurring only in Fos-expressing neurons of the dorsal striatum [248]. In these neurons, significant increase in mRNA levels of IEGs (Arc, Egr1), BDNF, and its receptor tropomyosin receptor kinase B (TrkB), as well as metabotropic GLUT receptor subunits (Gria1, Gria3, and Grm1), correlates with several epigenetic enzymes including KDMA1 [248] and HDAC5 [248, 249].

#### 4.3.3. METH and DNA Methylation

DNA methylation refers to the classic chemical covalent modification of DNA, which results from the addition of a methyl group at the 5′ position of a cytosine base via enzymes of the DNA (cytosine-5)-methyltransferases (DNMTs) family [264]. These include DNMT3A and DNMT3B, which are de novo methyltransferases, and DNMT1, that is, a maintenance methyltransferase [264]. This primarily occurs in DNA sequences where a cytosine (C) precedes a guanine (G) with the interposition of a phosphate group (CpG). CpG sites are unevenly distributed throughout the human genome both as interspersed CpG regions and as CpG clusters representing the so-called CpG islands. In line with the concept that promoters are the most sensitive to epigenetic changes, CpG islands occur mainly within promoter regions [265]. DNA hypermethylation of CpG within promoters represses transcription while DNA hypomethylation is often associated with increased gene expression [264]. It is worth mentioning that stability and activity of DNMTs depend on posttranslational mechanisms (phosphorylation, acetylation, and methylation) carried out by several kinases, such as CDK5 [266] and histone remodeling enzymes, especially HDACs [267]. In fact, in combination with increased HDACs, chronic METH reduces DNA methylation of the promoter region of GluA1 and GluA2 AMPAr subunit genes. This is confirmed by the finding that following chronic METH, there are decreases of 5′-methylcytosine (5mc) and 5′-hydroxymethylcytosine (5hmc) at the level of the promoter region of these genes [259]. At striatal level, METH-induced hypomethylation or hypermethylation may also affect corticosterone and glucocorticoid receptors' gene promoters [268, 269].

*(1) DNA Methylation in Human METH Abusers: The Convergent Role of DA and Oxidative Stress on Cell-Clearing Pathways and a-syn Expression*. Aspired by the vast body of evidence reporting aberrant promoter DNA methylation in psychotic disorders, a recent study investigated DNA methylation and gene expression pattern in human METH-induced psychosis [270]. RNA and DNA samples were extracted from the saliva of METH-addicted patients with and without psychosis, as well as from control subjects (each group *N* = 25). Despite carrying the inherent limit of a peripheral analysis, which may not be relevant for brain alterations, these findings demonstrate DNA hypomethylation within promoters of genes related to DA metabolism. In fact, DNA hypomethylation was present on the promoter of DRD3, DRD4, and membrane-bound catechol-O-methyltransferase (MB-COMT) genes. COMT provides a methylation of a hydroxyl group (which generates a methoxy group) of DA-forming 3-methoxytyramine (3-MT). Thus, DNA hypomethylation of MB-COMT gene promoter and increased COMT expression associate with synaptic DA degradation in the prefrontal cortex in psychotic METH abusers [270, 271]. Furthermore, DNA hypomethylation of AKT1 promoter gene was detected in METH patients with and without psychosis [270]. AKT1 gene encodes a serine/threonine kinase protein, which is expressed at high levels in the brain, and it is linked to DNA transcription, neural survival and growth, synaptic plasticity, and working memory [272, 273]. For instance, AKT regulates CREB- and NF*κ*B-dependent gene transcription [274, 275]. In addition, it phosphorylates DNMT1, thus playing a role in the switch between methylation, phosphorylation, and UPS-dependent degradation regulating DNMT1 stability and activity [276]. Remarkably, alterations of AKT levels and downstream pathways are closely related to the activity of DA receptors [277–280]. In line with this, dysregulation of AKT is reported in PD patients [281] and in METH experimental models [278]. Two downstream targets of AKT are glycogen synthase kinase 3 beta (GSK3*β*) and mammalian target of rapamycin (mTOR), a serine/threonine protein kinase complex. mTOR phosphorylates AKT via a feedback mechanism, while it activates p700Sk6 and 4EBP1 TFs. Once activated, TFs translocate in the nucleus to promote cell proliferation and survival. In line with this, inhibition of mTOR by the gold standard inhibitor rapamycin blocks drug-induced sensitization [282]. In contrast, mTOR activation inhibits ATG, which worsens METH toxicity [83, 283, 284]. In fact, prolonged METH exposure engulfs ATG machinery, which is upregulated as a compensatory mechanism [83, 86, 283, 284]. However, the bulk of oxidative species overwhelms the ATG machinery, which becomes progressively impaired as witnessed by the stagnant nature of ATG vacuoles suppressing the clearance of *α*-syn aggregates [83]. In line with this, an epigenetically induced upregulation of the *α*-syn coding gene SNCA was recently detected in the SN of rats exposed to METH [285], lending substance to an increase in *α*-syn protein levels [79]. Such an effect is associated with hypomethylation of the SNCA promoter, as shown by a decreased occupancy of MeCP2 and DNMT1 in such a region [285]. The effects of mTOR also relate to UP, which seems to be activated by mTOR inhibition [286–288] and inhibited during METH toxicity [38, 79–81, 289]. Noteworthy, the clearance of *α*-syn depends also on UP activity [79] and on a recently described ATG-UP merging organelle (the “autophagoproteasome”), which is directly activated by mTOR inhibition [287].

No study so far demonstrated an epigenetic regulation of SNCA within the striatum following METH; however, epigenetic modifications of SNCA have been documented in PD patients [290–292]. In fact, significant hypomethylation of CpG sites in the promoter region of SNCA is reported within leukocytes [292] and postmortem brain samples from patients with sporadic and complicated PD [290, 291, 293, 294].

#### 4.3.4. METH and DNA Hydroxymethylation

In recent years, DNA hydroxymethylation, generated by the oxidation of 5-methylcytosine (5mC) to 5-hydroxymethylcytosine (5hmC), became increasingly important in epigenetics [295]. It has been has suggested that 5hmC recruits DNA repair proteins and DNA demethylating machinery [295]. The formation of this modified base is mediated by ten-eleven translocation (TET) proteins and by TET-dependent generation of 5-formylcytosine and carboxyl-cytosine, which are then processed by thymine DNA glycosylase (TDG) and base excision repair (BER) mechanisms. The biological functions of 5hmC, which is highly enriched in the adult brain, appear to be crucial to promote gene expression related to quick behavioral adaptation [296]. Two recent studies demonstrated that compulsive METH intake is associated with large-scale changes in DNA hydroxymethylation in the rat NAc, which is consistent with a potential role for DNA hydroxymethylation in addiction [250, 251]. Remarkably, DNA hydroxymethylation around the transcriptional start site (TSS) or within intragenic regions of genes coding for neuropeptides was shown to occur following chronic METH administration [251]. This is the case of corticotrophin-releasing hormone/factor (Crh/Crf), arginine vasopressin (Avp) and cocaine- and amphetamine-regulated transcript propeptides (Cartpt), which increase in the NAc of METH-treated rats [251, 297]. In detail, Crh and Avp hydroxymethylation is mediated by TET1 and TET3 enzymes, respectively. In contrast, METH-induced changes in Cartpt expression derive from the binding of pCREB at the Cartpt promoter [251]. Together, these results support the hypothesis that METH produces a variety of epigenetic changes in the neuroendocrine circuitry within the NAc. This same epigenetic mechanism was recently studied within a context of compulsive METH intake [250]. It was found that in METH-addicted animals, which develop compulsive self-administration, hydroxymethylation occurs near or within genes coding for voltage-gated Ca+ channels. This occurs in different postsynaptic sites within the NAc, dorsal striatum, and prefrontal cortex of METH-addicted animals. Interestingly, hydroxymethylation of K+ channel-coding genes was found only within the NAc of nonaddicted animals [250].

## 5. Concluding Remarks

The influence of epigenetics in drug abuse provides a novel and deeper insight to understand the molecular mechanisms of addiction. This is key in the case of METH abuse since this drug possesses a variety of effects, which recapitulate the molecular alteration occurring in some neuropsychiatric disorders. As novel epigenetic changes are constantly being identified, it is more and more clear how simple effects induced by transient neurotransmitter alterations may translate into persistent alterations of brain physiology. Moreover, the multiplicity of findings revised here, when joined with a better knowledge of the genetic background, may clarify the interdependence between genetics and epigenetics underlying diversity in the human genome [298]. This leads to take into account the fact that a molecular cause-effect interplay between genetic and epigenetic factors during METH addiction may exist as well. Despite being yet unexplored in the context of drug abuse, such a close relationship is likely to explain the very peculiar phenotypic alterations observed during METH abuse. Such an intriguing issue surely deserves further attention and may represent a powerful tool for identifying additional genetic and epigenetic biomarkers to develop personalized treatments.

## Abbreviations

3-MT: 3-Methoxytyramine
5hmc: 5′-Hydroxymethylcytosine
5-HT: 5-Hydroxytryptamine (serotonin)
5mc: 5′-Hethylcytosine
Ac: Acetyl group
AC: Adenylate cyclase
AD: Aldehyde dehydrogenase
AKT1: Ak strain-transforming oncogene homologue serine/threonine kinase 1
AMPA: *α*-Amino-3-hydroxy-5-methyl-4-isoxazolepropionic acid
AMPAr: *α*-Amino-3-hydroxy-5-methyl-4-isoxazolepropionic acid receptor
AP-1: Activator protein 1
*α*-syn: Alpha-synuclein
ATF 1–4: Activating transcription factors 1–4
ATG: Autophagy
Avp: Arginine vasopressin
BBB: Blood-brain barrier
BDNF: Brain-derived neurotrophic factor
BER: Base excision repair
CaMKII: Calmodulin-dependent kinase II
cAMP: Cyclic adenosine monophosphate
Cartpt: Cocaine- and amphetamine-regulated transcript propeptides
CBP/p300: CREB-binding protein/p300 histone acetyltransferase
CBP: CREB-binding protein
Cck: Cholecystokinin
CDK5: Cyclin-dependent kinase 5
CNS: Central nervous system
COMT: Catechol-O-methyltransferase
COX-2: Cyclooxygenase-2
CpG: Dinucleotide composed of cytosine (C) preceding a guanine (G) with the interposition of a phosphate group (p)
CRE: cAMP-responsive element
CREB: cAMP response element-binding protein
Crf: Corticotrophin-releasing factor
Crh: Corticotrophin-releasing hormone
DA: Dopamine
DARPP-32: Dopamine- and cAMP-regulated phosphoprotein
DAT: Dopamine transporter
ΔFosB: Splice variant of the FBJ murine osteosarcoma viral (V-Fos) oncogene homolog protein B
DNMT: DNA (cytosine-5)-methyltransferase
DOPAC: 3,4-Dihydroxyphenylacetic acid
DOPALD: 3,4-Dihydroxyphenylacetaldehyde
DRD1–5: Dopamine receptors D1–D5
EAAT3: Excitatory amino acid transporter 3
Egr1–3: Early growth response proteins 1–3
Elk-1: Transcription factor of the E twenty-six-specific (ETS) domain family
ER: Endoplasmic reticulum
ERK1/2: Extracellular signal-regulated kinases 1/2
Fos: FBJ murine osteosarcoma viral (V-Fos) oncogene homolog transcription factor family containing cellular Fos (c-Fos), Fos proteins B and D (Fos-B and Fos-D), and Fos-related antigens 1 and 2 (Fra-1 and Fra-2)
Fst: Follistatin
GAD-67: Glutamate decarboxylase 1
GLUT: Glutamate
GluA1-2: Glutamate AMPA receptor subunits 1-2
GluN1: Glutamate NMDA receptor subunit 1
G_o/i_: Adenylyl cyclase inhibitor guanine nucleotide-binding protein
G_olf_: Olfactory guanine nucleotide-binding protein
G_s_: Adenylyl cyclase stimulatory guanine nucleotide-binding protein
GSK3*β*: Glycogen synthase kinase 3 beta
H: Histone
H_2_O_2_: Hydrogen peroxide
HAT: Histone acetyltransferase
HD: Huntington disease
HDAC1-11: Histone deacetylases 1–11
HSP70: Heat shock protein 70
IEGs: Immediate early genes
InhbA: Inhibin beta A
iNOS: Inducible nitric oxide synthase
IP3: Inositol 1,4,5 trisphosphate
Jun: Avian sarcoma virus (v-Jun) oncogene homolog transcription factor family containing cellular Jun (c-Jun) and Jun proteins B and D (Jun-B and Jun-D)
K: Lysine residue
KDMT: Histone lysine demethylase
KMT: Histone lysine methyltransferase
KO: Knock out
MAO-A: Monoamine oxidase type A
MAO-B: Monoamine oxidase type B
MAPK: Mitogen-activated protein kinases
MB-COMT: Membrane-bound catechol-O-methyltransferase
Me: Methyl group
METH: Methamphetamine
MLL1: Mixed lineage leukemia member of the KMT family
MSNs: Medium-sized spiny neurons
mTOR: Mammalian target of rapamycin
NAc: Nucleus accumbens
NE: Norepinephrine
NFAT: Nuclear factor of activated T-cells
NF*κ*B: Nuclear factor *κ*B
NMDA: N-Methyl-d-aspartate
NMDAr: N-Methyl-d-aspartate glutamate receptor
Nmu: Neuromedin U
NO: Nitric oxide
Nr4a: Nuclear receptor 4a
Nrf2: Nuclear factor erythroid 2- (NFE2-) related factor 2
Oxtr: Oxytocin receptor
pCREB: Phosphorylated CREB
PD: Parkinson's disease
PKA: Protein kinase A
PLC: Phospholipase C
PP1: Phosphatase protein 1
PP-2A: Phosphatase protein 2A
P-T34/75: Phosphorylated threonine 34/75
REST: Repressor element-1 silencing transcription factor
RNS: Reactive nitrogen species
ROS: Reactive oxygen species
SIRT: Sirtuin family of histone deacetylases
SNCA: Alpha-synuclein coding gene
SNpc: Substantia nigra pars compacta
STEP: STriatal-enriched protein tyrosine phosphatase
Syp: Synaptophysin
TDG: Thymine DNA glycosylase
TET: Ten-eleven translocation proteins
TF: Transcription factor
TH: Tyrosine hydroxylase
Thr34/75: Threonine 34/75
TrkB: Tropomyosin receptor kinase B
TSS: Transcriptional start site
UchL1: Ubiquitin C-terminal hydrolase L1
UP: Ubiquitin proteasome
UPS: Ubiquitin proteasome system
VGLUT-1: Vesicular glutamate transporter type-1
VMAT-1/-2: Vesicular monoamine transporter type-1/-2.
